# Religion, culture, and cancer: insights from a qualitative study on coping experiences of Filipino patients

**DOI:** 10.3389/fpsyg.2024.1457027

**Published:** 2024-09-06

**Authors:** Fereshteh Ahmadi, Saeid Zandi, Mae-Lanie Poblete

**Affiliations:** ^1^Department of Social Work and Criminology, Faculty of Health and Occupational Studies, University of Gävle, Gävle, Sweden; ^2^College of Health Sciences, Mindanao State University - Iligan Institute of Technology, Iligan City, Philippines

**Keywords:** cancer survivors, coping process, coping resources, coping strategies, faith, meaningmaking, religious coping, spirituality

## Abstract

**Introduction:**

Meaning-making coping is used by people with cancer to help them deal with the stress and emotional turmoil associated with their diagnosis. It is a multifaceted approach that can be influenced by cultural, existential, and personal factors. Research on meaning-making coping among Filipinos diagnosed with cancer is sparse. This study examines how a sample of Filipino people with cancer cope with their illnesses.

**Methods:**

We employed a qualitative approach. The study involved 20 participants with various types of cancer in the Philippines who were selected through purposive sampling and agreed to be interviewed.

**Results:**

The thematic analysis of the interviews revealed the application of both religious and secular meaning-making coping by participants. The study also confirmed the potential influence of culture on coping strategies.

**Discussion:**

The results indicate that religion plays a significant role in Filipino coping mechanisms, given that the Philippines is predominantly a Christian nation. Additionally, the findings highlight the importance of close family ties in Filipino culture and how it contributes to coping with cancer.

## 1 Introduction

Cancer is one of the leading causes of death in the Philippines, a country with over 113 million people (Dee et al., 2022). According to a study by the Institute of Human Genetics at the UP Manila National Institutes of Health, 189 out of every 100,000 Filipinos are diagnosed with cancer (Sung et al., 2021). Additionally, cancer claims the lives of four Filipinos every hour, totaling 96 deaths daily. In 2020, there were over 150,000 new cancer cases and 90,000 cancer-related deaths (Dee et al., 2022). During the first half of 2021, the Philippine Statistics Authority reported over 27,000 cancer deaths, accounting for 9% of all deaths in that period (UP Media and Public Relations Office, 2022). The five most common types of cancer in the country are breast cancer, lung cancer, colorectal cancer, liver cancer, and prostate cancer. The incidence of cancer in the Philippines is expected to rise in the coming years due to various factors, including an aging population, changing lifestyle habits, and environmental factors. The estimated number of new cases for 2030 is 239,114 (Bray et al., 2024).

For a person with cancer, the thought of having cancer can trigger anxiety and fears. Such fears may include concerns about the effects of the disease, the uncertainty of treatment outcomes, changes in economic situations, or the potential destruction of personal relationships (Vrinten et al., 2017). In such cases, coping behaviors become essential as efforts to restore and maintain balance in the lives of individuals with cancer (Ahmadi et al., 2018). The strategies individuals use when faced with illness, accidents, misfortune, and other challenges are influenced by cultural and historical constructs.

Culture plays a significant role in shaping an individual’s coping process when faced with stress, illness, or misfortune (Ahmadi, 2006; Ahmadi and Zandi, 2021; Cetrez et al., 2022; Goldblatt et al., 2013; Lemos et al., 2022; Zandi et al., 2023). Understanding cultural differences in coping can help healthcare providers provide appropriate support and care to cancer patients from diverse backgrounds. People with cancer often employ various coping methods to find meaning in their situation, including spiritual, religious, and existential approaches. According to Aldwin (2007), culture influences the stress and coping process in four distinct ways. Firstly, the cultural context determines the kinds of stressors an individual is likely to encounter. Secondly, culture impacts how stressful an event is perceived to be (Ji et al., 2022). Thirdly, cultural elements and norms guide the selection of coping strategies an individual employs in various situations (Cetrez et al., 2022; Khodayarifard et al., 2021). Lastly, culture offers diverse institutional mechanisms to support individuals in managing stress. These mechanisms may include social support networks, healthcare systems, or religious institutions that offer guidance and support during challenging times (Ahmadi et al., 2023; Zandi et al., 2023).

In this article, we aim to identify the coping methods used by a group of Filipino people with cancer to deal with their illnesses. We will also delve into the third way culture impacts coping, which is the role of culture in the selection of coping methods. Our focus will be on meaning-making coping methods.

### 1.1 Meaning-making coping

To adapt to the life situation, it is necessary to change one’s life goals and expectations. This is especially so when the disease often interferes with life goals and long-term life plans (Sloan et al., 2017). Individuals with cancer use to continuously search for the meaning of illness and self after diagnosis. This search for meaning may lead to a need to understand illness or give it meaning.

Coping in relation to find meaning has been shown to lead to positive effects (Folkman and Moskowitz, 2000). Meaning-making enriches positive emotions by helping individuals broaden their thoughts and actions to build up their patience to face negative emotions (Fredrickson, 2000). Meaning-making is an important factor for adaptation (Davis et al., 2000). Some researchers maintain that it even improves the mental health and well-being of patients (Martino and Freda, 2016; Park et al., 2008; Tolstikova et al., 2005).

Meaning-making coping refers to the process of finding or creating meaning and purpose in response to a stressful or challenging situation. This type of coping involves actively trying to understand and make sense of a difficult experience, rather than simply trying to avoid or eliminate it. For some individuals, their faith or spirituality can provide a source of comfort and help them find meaning in difficult experiences (Ahmadi et al., 2022; Ahmadi et al., 2018).

The process of meaning-making coping among cancer survivors consists of attempts to comprehend stressors caused by their illness. It involves efforts to reach a sense of coherence. To overcome the stress and the anxiety which are brought about by cancer, the cancer survivors try to reduce “the discrepancy between their situational meaning (appraisals, meaning-making, and meanings made) versus their global meaning (global beliefs and goals) of cancer” (Calimag, 2022, p. 149). It is in this regard that meaning-making coping methods play an important role in cancer survivors’ psychological well-being and adjustment. As Calimag (2022, p. 149) mentions:

*“Eventually, their psychological adjustment depends on the cancer survivors’ efforts at meaning-making, which may influence the extent to which they successfully make meaning from their experience and the meanings arrived at after the experience, that is posttraumatic growth, meaningful life, and re-stored beliefs in a just world.”*

This article is based on an international project in 10 countries. In all our studies, we have proceeded from Ahmadi’s definition of meaning-making coping (Ahmadi and Ahmadi, 2018, p. 23–25). In our international project, we have used the term meaning-making coping to refer to the entire range of religious, spiritual, and secular-existential coping methods.

### 1.2 The cultural and religious background of the Philippines

In a study concerning improving cancer care in the Philippines, Dee et al. (2022) focused on the need for deliberate and careful implementation of the national integrated cancer control act. They emphasize that.

*“Social and cultural determinants of health must also be considered, given varying levels of socioeconomic development, diverse cultures, and heterogeneous belief systems. These significantly impact the Filipino patient’s ability and willingness to understand, seek, and accept healthcare and modern medicine, the frameworks of which are heavily based on Western cultures and populations, and often come at high out-of-pocket costs.”*

The core values in ways of thinking of Filipinos are based on religion and also family and kinship obligations. According to Calimag (2022, p. 151), “Key concepts to consider in understanding the Filipino perspective on death and dying include cultural values and beliefs related to religion, family, and interpersonal harmony.” Such being the case, we regard it necessary give a very brief explanation of the cultural and religious background of Philippines.

The Philippines population is over 119 million, equivalent to 1.46% of the total world population (Worldometer, 2024). It is made up of ethnic groups with diverse cultural ethnicities. The Philippines is perhaps the most westernized among the Asian countries. Despite this, as Jia (2021, p. 339) mentions,

*“Religion in the Philippines is defined as spiritual beliefs from a culturally context held by Philippine citizens. Religion holds a central place in the life of the majority of Filipinos. It is central not as an abstract belief system, but rather as a host of experiences, rituals, ceremonies, and adjurations that provide continuity in life, cohesion in the community and moral purpose for existence.”*

The Philippines is unique among its neighbors in the Southeast Asian region in that the majority of Filipinos identify as Christian (92.5%). Catholicism is the dominant religion and the largest Christian denomination, with estimates of about 79.53% of the population belonging to this faith in the Philippines. Religion plays a significant role in the lives of many Filipinos and is often used as a coping mechanism in times of stress and difficulty (Del Castillo and Alino, 2020). This is perhaps because religion, particularly Catholicism, is deeply ingrained in Filipino culture and daily life. It shapes family values, traditions, and social norms (Pineda, 2024).

It is important to recognize that Filipino culture is deeply rooted in the belief in a parallel spirit world that, while invisible, has a profound influence on the visible, physical realm. This belief system is integral to understanding Filipino spirituality and its impact on daily life (Sitoy, 1985).

Filipinos hold that spirits, or “anito,” are omnipresent. These spirits range from high creator gods who are revered for their overarching influence to minor spirits that inhabit natural elements such as trees, rocks, and streams. This spiritual presence is not seen as distant or abstract but as an active and immediate part of the environment that interacts with and affects human life (Sitoy, 1985).

The pervasive nature of these beliefs fosters a strong conviction in life after death. In Filipino cosmology, the spirit world is perceived as an extension of the physical world, where one’s actions and rituals can influence their spiritual journey and afterlife. This perspective is reflected in various aspects of Filipino culture, including religious practices, ceremonies, and social norms.

For example, ancestral veneration is a significant practice, with rituals and offerings made to honor and appease spirits of ancestors. These rituals are believed to ensure the continued favor of spirits and to seek their guidance in daily life. Additionally, many Filipinos engage in practices intended to maintain harmony with the spirit world, such as performing rituals before embarking on significant life events or during times of crisis (Almocera, 2023).

This belief in a dynamic and interactive spirit world also influences attitudes toward death. Rather than viewing death as an end, many Filipinos see it as a transition to another phase of existence. This outlook is evident in funeral rites and commemorative practices that emphasize continuity and connection with the spiritual realm. The concept of an afterlife is not merely about individual salvation but is often seen as a continuation of communal relationships and responsibilities.

## 2 Methodology

In this study, a qualitative research design was applied as we aimed to identify the methods of meaning-making in coping with cancer from the perspective of patients and describe the role of culture in the coping process. Therefore, the research paradigm in our study is interpretive or constructivist. Here is why this paradigm is appropriate:

1. The article explores how Filipino patients find meaning in their illness experiences, which aligns with the interpretive paradigm’s emphasis on understanding the subjective experiences of individuals.
2. The research highlights the influence of cultural factors on coping strategies. This reflects the constructivist view that reality is socially constructed and varies according to cultural context.

Twenty participants were recruited using purposive sampling with the criteria being male or female, 18 years old and above, diagnosed with cancer, and willing to be interviewed. Most of the participants were recruited from the hospital while they were awaiting treatment or consultation. This verified their diagnoses. Some were from endorsements of colleagues who happen to be a friend, a relative, or a family member. Table 1 shows the demographic information of the informants. Participants’ type of cancer and stage of cancer are presented in Table 2.

**Table 1:** Demographics of the participants (*n* = 20).

Characteristics		N
Gender	Female	15
	Male	5
Age	35–40	1
	41–50	4
	51–60	9
	61–70	3
	71–80	3
Education	Elementary	2
	High School	2
	Undergraduates	13
	Postgraduates	3
Employment status	Businessman	3
	Housewife	2
	Private sector	4
	Government sector	4
	Nurse	2
	Educator	3
	Farmer/Fisherman	2

**Table 2:** Participants’ type of cancer and stage of illness.

Participant	Type of cancer	Stage
P1	Pancreatic Head Mass I	I
P2	Breast	III
P3	Colon	III-B
P4	Breast, Lobular Carcinoma	II-B
P5	Cervical	I
P6	Colon	III
P7	Breast (left)	III-A
P8	Uterine	I
P9	Lung cancer (right)	II
P10	Breast	II-B
P11	Breast (right)	II-A
P12	Colon	II-A – III-A
P13	Cervical	I
P14	Malignant ductal carcinoma	III
P15	Renal carcinoma	III
P16	Breast	IV
P17	Breast	II
P18	Prostate	II
P19	Chondrosarcoma	I
P20	Colon	IV

Participants of the study were mostly female. The youngest was 35 years old and the oldest was 78 years old. They came from different walks of life, with educational attainment varying from elementary graduate to a doctorate degree holder. The most frequent types of cancer among the respondents were breast cancer (35%) and colon cancer (20%). As to level of cancer, some of them were diagnosed early while others were in the late stage. Only a few were survivors. All of the participants came from the Christian faith.

This study has been checked and approved by the Swedish Ethical Review Authority (protocol no. 2015/126). Prior to the interview, the researcher sought informed consent of the participants. The participants were informed about the study and its objective before consent was obtained. They were also given the opportunity to ask questions and clarifications about the study and the interview. The study required an audio recording to assist with the accuracy of the responses; however, the participants had the right to refuse the audio-recording. The cooperation of the participants was completely voluntary and with consideration of their preferred schedule. They had the right to discontinue participation. Moreover, the participants’ identities were kept confidential. Instead of their actual names, numbers/letters were used during the transcription of the answers. A semi-structured interview guide (see Appendix A) derived from the Swedish study (Ahmadi, 2015) was used for data collection. The sets of questions were also translated into Filipino and Cebuano for better understanding during the interview. Interviews were conducted by a professor of sociology, and took about 30–90 min. The medium of communication used were English, Filipino, and Cebuano.

We analyzed the data using a thematic analysis method (Braun and Clarke, 2006). After transcribing and translating the interviews into English, each transcript was read and re-read in order to obtain a general sense of the meaning of the phenomenon. For each transcript, significant statements that pertain to the phenomenon under study were extracted. From these significant statements, meanings were formulated which were then sorted into categories, clusters of themes, and themes. The coding based on the themes found in the study used a template analysis style, a theory-driven analysis (Cash, 2018), while categorization of the themes was based on the results obtained in other studies in the project (Ahmadi, 2006, 2015; Ahmadi and Ahmadi, 2018). Coding continued until a high level of inter-rater agreement was reached (about 90%). After the coding process was complete, we established the fundamental characteristics of the methods the informants employed to cope with their cancer disease. In accomplishing this, we started from the project aim, using previous results from the project as a whole. As concerns religious coping, we started from the Five Key Religious Functions that constitute the basis of RCOPE (Pargament et al., 2000). Finally, validation of the findings was sought from the research participants to compare the researcher’s descriptive results with their experiences.

## 3 Results

From the narratives, we found both religious meaning-making coping styles and non-religious meaning-making coping strategies. In the following, these methods will be presented.

### 3.1 Religious meaning-making coping

This study identified several RCOPE coping methods (Pargament et al., 2000) and two religious coping methods that were not included in the RCOPE list. These two methods, *Faith in Suffering: the Story of Job* and *Hope for Life After Death*, were reported by our study participants. In the following sections, we will present all these methods using direct quotes from the interviews.

#### 3.1.1 Religious methods of coping to find meaning

***Benevolent Religious Reappraisal***: redefining the stressor through religion as benevolent and potentially beneficial. Concerning this method, a woman, 35 years old, explained that:

*Even this cancer can be a blessing in disguise because it helped me to be stronger and to have a spiritual awakening as I continue to serve Him and God’s people. Having constant communication with God is important.*

#### 3.1.2 Religious methods of coping to gain control

***Active Religious Surrender***: Both a 57-year-old woman and man described how they actively gave up control to God and used prayer as their coping method:

*So when I learned about my illness, I did not blame God because I believed that there’s a higher purpose behind all of these. I just must accept it because God will not abandon me. For me, the most important is my faith in God.*

*At first, I just tried to ignore my symptoms and pray that they will just go away but, it got worsened. I feel anxious as the days passed by waiting for the results. The truth hurt me but it did not quench my spirit. I may not be that religious, but I still believe in a personal relationship with God. Even if the doctors presented options for me but ultimately, I must decide for my health and any treatment available. I tried to play smart and think about what was best for me. But deep inside, I am screaming for god’s help and power in my life. In the end, I fixed my eyes on my Creator and surrendered everything to Him. Let thy will be done in my life.*

***Passive Religious Deferral***: Passive waiting for God to control the situation. During our research, we spoke with several informants, including one middle-aged woman and two older adults (one male and one female), who spoke to us about their passive approach to waiting for a change in their disease situation.

*I welcome death because I believe in life after death. I welcome it to end my pain and suffering.*

*I do not worry about tomorrow. I am confident that God has a purpose for my life. God will take care of me and my family.*

*I gained a deeper understanding of my faith and beliefs. Whether I am sick or not, my faith in God is the same because I am serving the One and Only God who never changes. I surrender my life to Him. From the start, I offer it to God and continue to trust Him.*

***Pleading for Direct Intercession***: Seeking control indirectly by pleading to God/ a Spiritual Being for a miracle or divine intercession. A 49-year-old woman and a 51-year-old man explained how they prayed for a miracle:

*There is nothing I can do but pray. Praying for healing, for comfort, for God’s miracle. I pray all the time and hope that God will hear my prayer.*

*I admit even to this point, I asked God to give me a miracle. I believe that nothing is impossible in Him.*

#### 3.1.3 Religious methods of coping to gain intimacy with others

***Religious help***: Two women, one aged 57 and the other 62, explained that they sought the help of others through prayer, as indicated in the citations below:

*I feel loved when people pray for me, for my healing.*

*Also being a part of a religious community helped me spiritually. I find support through them, and it feels good knowing that other people are praying for me.*

#### 3.1.4 Religious methods of coping to achieve a life transformation

***Seeking Religious Direction***: A 58-year-old man described how he turned to religion for guidance when his old way of life was no longer viable due to cancer. He found a new direction in life after this experience:

*Before, material wealth and social stature were important to me. But now, I fully understood the power of having a strong and close family and the power of prayer in one’s life. I learned that the most important in life is a real relationship with God and family. Now, I value my wife and my family the most.*

***Religious Conversion***: A 53-year-old woman spoke about how she sought a radical change in life through religion:

*I am religious because I grew up in a Catholic household. We always went to church as a family and observed all rituals and teachings set by the Catholic church. However, I did not have a personal relationship with God until I was diagnosed with breast cancer. It was a turning point in my life. I cried so much. I never thought I can cling to God like that. I entrust to Him my life and now I feel so close to Him. Right now, I do not take for granted all that He has done for me. I feel His presence in my life, and I am never the same again.*

#### 3.1.5 Religious methods of coping to gain comfort and closeness to god

***Seeking Spiritual Support***: Several participants, including a 78-year-old man, a 64-year-old woman, and a 61-year-old man, shared their search for comfort and reassurance through God’s love and care:

*I admit when I was younger, I was just encouraged by my mother to go to Church and worship God. I meant I had no deep personal relationship with God. …. As I became, sick and experienced hopelessness in my body, the more I talked to Him and cry out my apprehensions and fears. I believed that God wants us to understand not by mere thoughts and habits, but by having a real relationship with Him. I guess that is what’s different now that I am aging than before when I was young and active…Now I understand what it meant by “apart from God, I am nothing.”*

*My relationship with God and constant praying helped me cope with cancer. I pray all the time. I make it a point to have personal time and devotion to God. I meditate upon His words and enjoy singing praises He truly deserves. I still serve in church and going to church is still a must for me and my entire family.*

*I give importance to my relationship with God the most. My faith in Him was strengthened when He healed me from cancer. It was my faith in Him that helped me deal with cancer …God never abandoned me and will never abandon me. I continue to entrust to Him my life.*

***Religious Focus***: Two women, one 50 years old and the other 61 years old, described their engaging in religious activities to shift focus from the stressor:

*I still perform my religious activities every Sunday and Wednesday because it is my novena to Perpetual Help.*

*During the time I had cancer, I continue to nurture my faith through prayer. I also went to different churches and participated in healing masses. I considered myself a fighter for my family. I grab every opportunity I can get to be healed. I can say that I am also a prayer warrior. Knowing that other people were also praying for my healing refreshes me.*

***Religious Purification***: A 50-year-old woman gave an explanation about her search for spiritual cleansing and forgiveness through religious actions:

*When I pray, I adore God first, and then ask for forgiveness, followed by thanksgiving and asking for healing of my body, soul, and spirit.*

***Spiritual Connection***: A 64-year-old woman told us about experiencing a sense of connectedness with God:

*My relationship with God and constant praying helped me cope with cancer. I pray all the time. I make it a point to have personal time and devotion to God. I meditate upon His words and enjoy singing praises He truly deserves.*

Our informants also used two religious coping methods which were not on the list of RCOPE.

#### 3.1.6 Faith in suffering: the story of job

The story of Job in the Bible is a profound illustration of how religious faith can be a source of comfort and resilience in the midst of great suffering. Job was a righteous man who faced unimaginable loss and affliction, including the deaths of his children, the loss of his wealth, and a severe deterioration in his health. Despite these trials, he remained steadfast in his devotion to God and refused to curse Him, even when his wife and friends encouraged him to do so.

A woman, 72 year old, explained the use of the religious coping “faith in suffering” by referring to Job as follows:

*During my surgery, in the operating room, I thought I would die but God did not take me yet. This is a test of faith, just like Job.*

#### 3.1.7 Hope for life after death

The coping method “hope for life after death” is a belief held by some people that there is an afterlife or a continuation of the soul beyond physical death. This belief can provide comfort and solace to those who are facing the loss of a loved one or their own mortality. It can also help individuals find meaning and purpose in their suffering and provide a sense of hope that their pain and struggles in this life will be rewarded in the next. For some, it may simply be the hope that there is something more beyond this life and that death is not the end. This belief can also offer a sense of peace and acceptance, as it allows individuals to view death as a natural part of the cycle of life rather than an ultimate end. This coping mechanism may not be effective for everyone, it can be a powerful source of comfort and can offer a way to endure the challenges of life with a sense of purpose and meaning for some, and provide a sense of hope and comfort in the face of tragedy and loss.

Several participants, women and men, explained how this idea has helped them to cope with their illness:

*I am trying to stay positive despite the situation. I believe that there is life after death and God has prepared a wonderful place for me in his kingdom. There is life after death. The promise of heaven is my hope. (F, 50).*

*Now, I have a positive view of life after death. It is not “death per se” but another new life – a life with God, spending eternity with Him. (F, 53).*

*I always believe in life after death. I have accepted Christ as my personal Lord and Savior and I hope someday He will welcome me into His kingdom. (M, 57).*

### 3.2 Secular meaning-making coping

Secular meaning-making refers to the process of finding purpose and meaning in life without relying on religious or spiritual beliefs (Zandi et al., 2023). This can involve finding meaning through personal growth, relationships, work, creativity, or other sources of fulfillment. In the context of coping with serious illnesses such as cancer, secular meaning-making may involve finding purpose and meaning through activities such as advocacy, volunteering, or participating in support groups (Ahmadi et al., 2022; Ahmadi et al., 2018). It may also involve finding new sources of fulfillment and purpose in life, such as pursuing creative hobbies or taking on new challenges. It is important to note that secular meaning-making is not mutually exclusive to religious or spiritual meaning-making, and many individuals may use a combination of these approaches to find purpose and meaning in their lives. Additionally, the effectiveness of different meaning-making strategies may vary depending on individual factors such as personality, culture, and social support networks.

In this study, we identified certain coping methods which can be characterized as secular meaning-making coping, these are family and friends, and to be positive and live in peace.

#### 3.2.1 Friends and family

The meaning-making coping strategy “family and friends” was one of those meaning-making coping methods that several participants mentioned as an important coping method in dealing with the psychological challenges brought about by cancer:

*I am thankful that my family never gave up on me. They were the ones who comforted me during those difficult times when all I wanted was to stay in my room and cry. I feel so loved by their encouraging words and for letting me know that there is still hope. This is not the end of the world for me. For now, my world is my family. (F, 49).*

*I appreciate when my wife and kids take care of me, our talks, and gatherings. They give me peace and satisfaction along with my wonderful grandchildren. (M, 56).*

*I value my family, my wife, my children, and my grandchildren. They helped me to manage my disease. They are my source of inspiration and strength. I love them so much. (M, 78).*

*My family is my anchor. I cannot imagine my life without them. I love them so much. They assisted me in everything that I do and they never get tired to show me how much they appreciate me as a mother and wife. I am blessed to have them in my life. (F, 58).*

*I am forever grateful for my family and the fact that we are not having problems when it comes to relationships because we understand each other. My family, especially my children, have been very supportive of me financially and emotionally. (F, 72).*

*Having a good and harmonious relationship with my family gives me peace and satisfaction. My strong family ties have been instrumental during this crisis. It feels good to be appreciated by friends; but most of all, I love to spend my precious time with my immediate family, especially my grandchildren. They are my source of joy. (F, 64).*

Even those who do not have their own family admit that their friends and their families have played a role in their coping with their disease.

*Even if I have no family of my own, I am thankful to have the support of my friends and immediate family. Life is hard but it becomes bearable knowing that there are people who care for you. (M, 76).*

*I do not know if I can manage on my own without my family. It is difficult but I am thankful to have my family who loves me and understands me. I am also grateful to my friends and co-workers, even my students for their understanding. I am a teacher and I wanted to be surrounded by people. It seems that I get my energy from them as well. I love to spend time with my friends. (F, 53).*

#### 3.2.2 To be positive and live in peace

The participants also employed the coping method “To be positive and live in peace.” Three women, 45 years old, 57 years old, and 61 years old have used this strategy:

*Aside from God and my family, I made it because I stayed positive. I did not allow cancer to ruin me. I keep my perspectives and views in life positive by looking into the good things in life and counting my blessings. (F, 45).*

*For me, it’s important to think positively. We are all but passers in this world. That’s why it’s important to value our relationships. Death is not something to be fearful of but rather we must prepare for it. (F, 57).*

*The second most important thing in my life is my relationship with my fellowmen. As best as I could, I want to live in peace with others. (F, 61).*

## 4 Discussion

The current study aimed to identify the meaning-focused coping methods used by Filipino patients to deal with their illness. Our findings showed that our Filipino respondents turn to their religious beliefs and practices for comfort and guidance during times of crisis. Religious coping is prevalent in the Philippine. It is used during natural disasters such as typhoons or earthquakes; people often come together to pray for safety and protection. In times of personal struggle, Filipinos may turn to prayer and meditation as a way to find inner strength and peace (Bulisig and Aruta, 2022; Canete, 2021; Manalo et al., 2023). Religious organizations in the Philippines also play a crucial role in providing aid and support to those in need (Tan, 2023). Churches and other religious groups often organize relief efforts and provide material and emotional support to those affected by disasters, poverty, or other challenges. Overall, religion plays a significant role in the coping strategies of many Filipinos. It provides a sense of community, comfort, and hope during difficult times and offers a framework for understanding and making sense of the world (Dodd et al., 2023).

Some studies (Canete, 2021; Doorenbos et al., 2011; Lagman et al., 2014; Miranda et al., 1998) showed that those who were strict in their religious beliefs and practices tended to attribute illness to the will of God or a higher power, even if they also believed in personal responsibility. Their faith and hope in God’s plan for their lives, their attitude to God’s providence, their often fatalistic and deterministic approach to life have caused them to give substance to the physical pain and sense of emptiness caused by their illness.

Filipinos hold a strong belief in God’s will, and while their unwavering faith has often been positive, in certain situations, it has transformed into a significant issue, impacting not only individuals but also society as a whole. One example explained by Canete (2021, p. 1) is as follows:

*“The Filipino people are known for their strong religious faith. Even in the midst of the most trying moments of their collective history, their faith remains steadfast. The isolation brought by the COVID-19 pandemic did not stop the Filipino Catholic faithful from expressing their faith but made it stronger. Allowed by the government to attend Church celebrations, the Filipino Catholic devotee flocked in the Quiapo Church just to attend the feast of the Black Nazarene and had a chance to hold its image with a firm conviction that the grace of God could protect them from the pandemic. However, the local medical experts called their devotion, in this time of crisis, as a ‘superspreader’, a sign not of hope but of despair.”*

One of the new religious coping methods we found in this study was “hope for life after death.” As mentioned in the section “The cultural and religious background of the Philippines,” one of the features of the Filipinos’ religious ways of thinking is the belief in a parallel spirit world and the omnipresence of spirits deeply shapes Filipino cultural practices and worldviews. It creates a framework where the spiritual and physical realms are interwoven, influencing not only religious practices but also everyday interactions and attitudes toward life and death. Indeed, the belief in a pervasive spiritual presence contributes to a strong conviction in life after death among Filipinos.

Our findings indicated that family serves as a coping resource for people with cancer in Philippines. Although the family is an important institution in many societies, its central role in the lives of family members in the Philippines is, as some researchers (Calimag, 2022; Virola et al., 2012) maintain, unusually significant. Family is rated being the most essential source of happiness in this country (Virola et al., 2012). Family is the basic social and economic unit of Filipino kinship (Calimag, 2022). The family unit in the Philippines is often characterized by strong bonds between relatives, a sense of loyalty, and a deep sense of responsibility toward one another.

Filipinos typically have large families, and the concept of “extended family” is widely recognized. This means that grandparents, aunts, uncles, and cousins are often seen as immediate family members and are given the same level of care and respect as parents and siblings. Family plays a central role in the social and cultural fabric of the Philippines. It provides a sense of belonging, social support, and financial stability, and is considered a cornerstone of Filipino society (Choi et al., 2018; Gozum, 2020).

One of the primary roles of the family in the Philippines is to provide financial support (Alampay and Jocson, 2011). It is common for children to provide financial assistance to their parents and grandparents, particularly in their old age. This tradition is known as “pagpapakatao” or “showing respect,” and it is seen as a way of honoring one’s parents and the sacrifices they made for the family. Another important role of the family in the Philippines is to provide emotional and social support (Javier et al., 2018). Families often come together to celebrate milestones, share meals, and participate in community events. They also provide a safety net during times of crisis, such as during natural disasters or economic hardship.

In times of crisis, especially serious illness, the family_ even the extended family_ provides support and assistance. Here cultural values play an important role. Calimag (2022, p. 152) emphasizes that.

“*Filipino values refer to the set of values that a majority of the Filipinos have historically held important in their lives. This Philippine values system includes their own unique assemblage of consistent ideologies, moral codes, ethical practices, etiquette, and cultural and personal values that are promoted by their society.*”

Family is a source of meaning-making in the Philippines. The concept of “pakikipagkapwa” or “concern for others” is an important value in Filipino culture. This value emphasizes the importance of maintaining harmonious relationships with others and treating others with respect and compassion. Family members often turn to each other to find meaning in the face of adversity, and they work together to find solutions to problems. Family plays a significant role in helping individuals in the Philippines cope with stress and find meaning in life. The close-knit nature of Filipino families and the emphasis on social support and pakikipagkapwa help individuals to weather life’s challenges with the help of their loved ones.

The findings of this study also demonstrated that positive attitude functions as a secular method of coping with cancer among the study participants. The ethos of being positive is a prominent feature of Filipino culture. Filipinos are known for their resilient and optimistic spirit, even in the face of adversity. One of the manifestations of Filipino positivity is the concept of “pakikisama,” which refers to the importance of getting along with others and maintaining harmonious relationships (Villero et al., 2014). Filipinos place a high value on social connections and often prioritize the needs and interests of the group over their own individual desires. This can lead to a strong sense of community and a willingness to work together to overcome challenges (Martinez, 2023). Filipinos are also known for their hospitality and generosity, which reflect a desire to make others feel welcome and comfortable. It is not uncommon for Filipinos to go out of their way to help others, even strangers, and to share what they have with those in need.

## 5 Conclusion

Summing up, the article discusses coping strategies for individuals with cancer in the Philippines, highlighting the importance of faith, spirituality, and emotional support from family and friends. Despite the challenges, many patients are able to maintain a positive and fulfilling lifestyle with the help of these coping mechanisms and supportive care. The article also notes that the culture of being positive in the Philippines reflects resilience, faith, and a strong sense of community among the Filipino people. The study also showed the role of culture in coping. Culture can influence an individual’s beliefs and values, which can impact their resilience and coping skills. Beliefs about the nature of illness, death, and suffering, for instance, can vary across cultures and shape an individual’s coping process. In the context of cancer, understanding the influence of culture on coping strategies is important for healthcare providers. By recognizing the role of culture in shaping coping methods, healthcare providers can tailor interventions to better support patients’ cultural needs and preferences.

### 5.1 Strengths and limitations of this study

The current study illuminates how our sample of Filipino people with cancer manage and endure their conditions. It also aims to enhance our understanding of the potential influence of culture on the use of various religious, spiritual, and secular existential coping strategies. By presenting and discussing our findings, we aspire to contribute to the expansion of scientific knowledge in the fields of meaning and coping. However, one limitation of this study is the sampling bias, as our sample was predominantly female. Therefore, we should be aware of the effect of gender but we can hardly explain this effect on the result. Additionally, we did not address patients’ biopsychosocial distress, which may impact coping mechanisms.

### 5.2 Implications for research and practice

Conducting similar research on coping mechanisms among various groups with different chronic illnesses can facilitate intergroup comparisons of result patterns. This approach may yield valuable insights into the interaction between humans and health crises. Future research could utilize nationwide or large-scale quantitative surveys to explore the prevalence of religious, spiritual, and secular existential coping methods identified in this study within the Philippines. Cross-tabulations based on sociodemographic factors (such as gender, age, and education) and clinical characteristics (such as the phase of illness) will enhance the informativeness of these quantitative efforts. It is crucial to consider the respondents’ phase of illness and survivorship in future studies on meaning-making coping processes, as cancer survivorship spans a continuum from diagnosis through treatment and beyond, with survivors’ experiences evolving across this spectrum. For a more thorough examination of coping patterns in patients, we recommend that future researchers also consider the biopsychosocial states of informants.

We advise health psychologists, psycho-oncologists, physicians, anticancer therapists, social workers, palliative care professionals, hospice care providers, and nurses involved in cancer care to utilize the current findings when advising Filipino clients with cancer. We also encourage health administration officials and cancer support service providers to offer evidence-based educational classes and self-study materials to inform people with cancer about potential coping strategies. Additionally, patients and survivors are encouraged to adopt a broader range of adaptive coping strategies to manage their illness and distress more effectively, thereby facilitating their adjustment to cancer.

## Data availability statement

The raw data supporting the conclusions of this article will be made available by the authors, without undue reservation.

## Ethics statement

The studies involving humans were approved by the Swedish Ethical Review Authority, Uppsala, Sweden (Decision No.: 2015/126). The studies were conducted in accordance with the local legislation and institutional requirements. The participants provided their written informed consent to participate in this study.

## Author contributions

FA: Formal analysis, Methodology, Writing – original draft, Writing – review & editing, Conceptualization, Funding acquisition, Investigation, Project administration. SZ: Formal analysis, Methodology, Writing – original draft, Writing – review & editing, Resources. M-LP: Data curation, Formal analysis, Writing – original draft, Writing – review & editing.
