# Supplementary material for: Religion, culture, and cancer: insights from a qualitative study on coping experiences of Filipino patients
Source: Front Psychol. 2024 Sep 6;15:1457027. doi: 10.3389/fpsyg.2024.1457027 (PMC11412877; doi:10.3389/fpsyg.2024.1457027)
Supplement: Supplementary file 1 [file Table_1.docx]

**Appendix A**

| ***Interview Guide***  [Introduction: Explanation about the aims of the interview]  1. Please share how you discovered that you had cancer.  a. Did you undergo any oncological treatments? [optional question]  b. Have you experienced any relapse? [optional question]  c. Did you explore non-conventional treatments? [optional question]  2. How did you handle the cancer diagnosis?  3. During your illness, what situations made it challenging to adapt to cancer?  4. People employ various strategies to cope with the disease. In your case, where did you find the strength to overcome the most difficult moments?  a. What role did family and friends play in how you handled cancer?  i. Who were the most important individuals in this process, and how did they support you?  b. How did religion influence the way you coped with cancer?  i. How did your spiritual beliefs help or hinder you during the disease?  ii. Overall, do you believe religion plays a significant role in your life?  iii. How does religion and praying impact your daily functioning?  iiii. Have your spiritual beliefs and religious practices changed after facing cancer?  5. Focusing on a broader dimension of spirituality, have you ever experienced a heightened sense of spirituality? [optional question – information could appear in the answer to question 4]  a. How did spirituality help or hinder you during the disease? [optional question – information could appear in the answer to question 4]  6. For some individuals, the relationship with nature serves as a crucial resource to deal with cancer. Has nature been an important resource for you in dealing with your disease? If yes, in what way?  a. Do you engage in regular meditation to cope with your illness?  7. With the cancer diagnosis, it is possible that the sense of life has changed.  a. How did you begin to handle day-to-day life after the disease?  b. Has being alone and contemplating your life and its meaning been a way to deal with your disease?  8. Sometimes, when people receive the diagnosis of cancer, they create theories regarding what may have caused their disease. What are your thoughts on this?  a. Have you ever wondered, "Why have I received a cancer diagnosis?"  b. Did you feel guilty for behaviors in the past? [optional question]  c. Have you ever thought that God or a spiritual being allowed this event to happen to you? [optional question]  d. Did you think that your illness is caused by an evil power? [optional question]  e. During the disease, have you ever felt abandoned by God? [optional question]  9. In general, when you feel nervous or concerned about something, what helps you to calm down?  a. How do you typically support others? |
| --- |
